# Lumbar Radiculitis as a Complication of Vaccination against Tick-Borne Encephalitis: A Differential Diagnosis of Low Back Pain and Nerve Root Compression

**DOI:** 10.1155/2020/6130364

**Published:** 2020-03-23

**Authors:** Craig Kingston, Günther Zech, Caroline Pauli, Ulrich Walker

**Affiliations:** ^1^Basel University Hospital, Department of Rheumatology, Basel, Switzerland; ^2^Praxis Pauli AG, Reinach, Switzerland

## Abstract

Serious adverse reactions following immunisation with adult tick-borne encephalitis (TBE) vaccines are rare, but when they occur, they most frequently involve the nervous system. We present a case of a female patient who developed a sensory and motor L4 monoradiculopathy following self-injection of an inactivated vaccine against TBE in the ipsilateral quadriceps muscle. The motor and sensory L4 dysfunction vanished after 12 months. TBE vaccine-induced radiculopathy should be considered as a mimic of spinal root compression.

## 1. Introduction

The TBE virus is a member of the genus *Flavivirus*, family Flaviviridae, and is the causative agent implicated in the disease TBE. In Western Europe, it is primarily transmitted via infected *Ixodes ricinus* ticks and has in recent years been observed at increasingly higher latitudes [1]. The total number of annual cases is estimated to be up to 13,000 in the Eurasian northern hemisphere [2].

In addition to physical barrier measures such as protective clothing, vaccination is the most effective method for preventing TBE. Two preparations are available for adults in Europe, namely, FSME-Immune™ and Encepur™. These vaccines have been robustly tested and are considered to be safe and effective [1].

Side effects are common but generally mild and transient and include mainly headache (12%), fever (1–10%), muscle pain (12%), and local irritation (46%). Transient paraesthesia occurs rarely (<0.01%). Isolated cases of serious adverse neurological events, including transient encephalitis, myelitis, oculomotor paresis, and Guillain–Barré syndrome, have been reported [3].

The following case describes a unique neurological side effect simulating a unilateral lumbar radiculopathy.

## 2. Case Description

A 40-year-old female patient presented to our service with a 5-day history of progressive right-sided low back pain, which radiated over the right buttock into the anterior thigh in an L4 distribution. The pain was nonpositional and exacerbated by coughing. She also complained of new bifrontal headaches, difficulty in concentrating, stool retention, and difficulty in passing urine. Two weeks previously, she had commenced an immunisation against adult tick-borne encephalitis (TBE) by self-injecting a vaccine (Encepur™) into the ipsilateral quadriceps muscle. Her medical history was otherwise unremarkable. Symptoms of a viral infection such as fever, rash, and oral or genital lesions were not present, and recent exposure to tics was denied.

The patient was afebrile, her blood pressure was 170/110, and her heart rate was 92. The alignment of the spinal column was normal. On the right leg, there was a positive straight-leg raising test, weakness of knee extension, and a diminished patellar reflex. There was also slight hypaesthesia over the knee and the anterior aspect of the thigh. The Babinski sign was negative, and there was no evidence of meningism such as photophobia, neck-stiffness, or a positive Brudzinski sign.

The ESR was 7 mm/hr, her CRP was 8.9 mg/L, and the blood differential was normal. The remaining blood chemistry was negative, including tests for paraproteins, ANA, and HIV. Abdominal ultrasound excluded urine retention and other intraabdominal pathology. On contrast-enhanced MRI, there was no compression of the spinal roots or the cauda equina and no involvement of the cranial meninges. The patient refused a lumbar puncture.

We diagnosed a sensorimotor radiculitis of the fourth lumbar root secondary to TBE vaccination. A 5-day course of prednisolone (1 mg/kg) did not influence symptoms and was thus discontinued. Three months later, the patient still required analgesics (NSAIDS, tramadol, and pregabalin) and had developed a pronounced weakness and atrophy of the quadriceps muscle and a foot drop of the affected leg. The motor and sensory function had started to return to normal at 6 months, and by 12 months, the deficit had vanished. In contrast to this, the headaches and difficulties to concentrate persisted at the one-year mark.

## 3. Discussion

According to the latest consensus, TBE vaccination is recommended for all age groups above 1 year in highly endemic areas (≥5 cases/100,000/year) and also for individuals at risk in areas with a lower incidence [2].

Many European countries are reporting an increasing incidence of TBE, the highest being in Slovenia, Lithuania, and Latvia [2] with foci now occurring as far north as Norway, Sweden, and Finland [4]. New endemic foci have recently been documented in Siberia, Mongolia, northern China, the Korean peninsula, Kyrgyzstan, Armenia, Azerbaijan, Uzbekistan, and Kazakhstan, including areas at altitudes of up to 2000 m. Austria is one of the few countries which has recently seen a fall in TBE cases due to a robust vaccination program, but the risk for the unvaccinated remains high [2].

**Figure 1:**
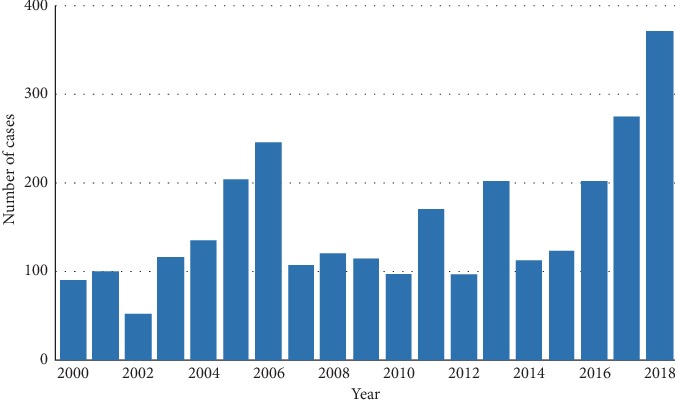
Annual number of TBE cases in Switzerland, adapted from [5] with permission.

The prevalence of infected ticks can also vary greatly within countries, with certain areas of Switzerland having a prevalence of over 14% in a single focus [1]. This trend towards an increasing number of cases per year is typical for many northern European countries. Until 2019, vaccination was only indicated for specific endemic regions within Switzerland but due to increasing incidence (see Figure 1), there is now a recommendation to vaccinate the population in the whole country, except the counties of Geneva and Tessin [5]. The seroprevalence rate in high-risk areas of Western European countries such as France remain however lower than those of other tick-borne diseases such as borreliosis or tularaemia [6].

The vaccine is normally administered in 3 doses over a period of 6 months. A Cochrane review summarized 11 vaccine trials, and amongst them, four randomized studies were on licensed European vaccines (Encepur and FSME-Immune) with a total of 5063 participants: the vaccines reached seroconversion rates of 92%–100% [7]. Cost-effectiveness studies on TBE vaccines have been performed in Slovenia, Sweden, Finland, and Estonia, demonstrating economic saving related to decreased incidence, especially of severe cases [8]. In vivo and in vitro studies have shown that the European vaccines protect against all TBEV subtypes circulating in endemic areas of Europe and Asia, indicating equally potent cross-protection [2]. Due to a decline in antibody production following vaccination in old age, an additional priming dose of the vaccine has been implemented in Sweden in those over 60 years of age [9].

Serious adverse reactions are extremely rare and most frequently involve the nervous system. The mechanism by which the vaccine may induce neurological deficits is not well understood. The most likely mode of damage is molecular mimicry, in which a conformational homology between a vaccine antigen and self-antigen directs T cells and/or humoral immunity towards host tissue. Molecular mimicry has been demonstrated in multiple animal models and most notably in experimental allergic encephalomyelitis [10]. Both TBE vaccine preparations have been implicated in nervous system adverse reactions [11, 12]. Swissmedic (formerly the Swiss Drug Monitoring Centre) received notifications between 1987 and 2000 regarding 33 patients with 39 adverse neurological reactions following TBE vaccination. Twelve of these patients were hospitalised, and all of the reactions were completely reversible after a maximal period of one month. The latency period ranged from a few hours to 30 days, with the majority of reactions after the first vaccination. This included three cases with radicular symptoms and five cases with meningeal symptoms [12].

In 2004, a case report of acute disseminated encephalomyelitis (ADEM) following the third vaccination against TBE was published from Germany. ADEM manifested as recurrent retrobulbar neuritis, grand mal seizures, and a single focal tonic-clonic episode. The causality was interpreted as “definite” by the author. A similar case was presented as a poster at the annual conference of the European Society of Neurology in 1998 [13]. In 1998, there was another case report from Germany of diverse psychomotor deficits (left faciobrachial sensory deficits, a fluctuating slowing of psychomotor function, disorientation, and memory deficits) in a patient following TBE vaccination, all of which had completely subsided 8 weeks after exposition [14]. From a publication in 1992, there were a total of 72 registered cases of neurological side effects following TBE vaccination [15]. From these, only three were considered definite or probable, and all presented as some form of neuritis without permanent sequelae. In a small randomized controlled trial of patients with multiple sclerosis, however, there was no significant worsening of symptoms of demyelination in the vaccinated group in comparison to the placebo group [16].

As these case reports show, motor, sensory, and memory deficits are known but are rare complications of vaccination. Our case is the first to demonstrate a prolonged neurological deficit as a result of TBE vaccination. Adverse reactions to TBE vaccines may be an underdiagnosed mimic of spinal root compression.

## 4. Conclusions

Neurological complications are one of the more common side effects of TBE vaccines, though most take the form of transient headaches. Serious neurological complications are exceedingly rare and to date, completely reversible. Tick-borne encephalitis vaccines can cause a radiculopathy that mimics spinal root compression, which can cause long-term functional deficits. The safety profile of the available TBE vaccines remains however excellent, and TBE vaccines should therefore continue to be administered in endemic areas, as this is associated with a dramatic fall in the number of TBE cases [17].
